# Genome-Wide Association Mapping Uncovers *Fw1*, a Dominant Gene Conferring Resistance to Fusarium Wilt in Strawberry

**DOI:** 10.1534/g3.118.200129

**Published:** 2018-03-30

**Authors:** Dominique D. A. Pincot, Thomas J. Poorten, Michael A. Hardigan, Julia M. Harshman, Charlotte B. Acharya, Glenn S. Cole, Thomas R. Gordon, Michelle Stueven, Patrick P. Edger, Steven J. Knapp

**Affiliations:** * Department of Plant Sciences; † Department of Plant Pathology, University of California, Davis, California, 95616; ‡ Department of Horticulture, Michigan State University, East Lansing, Michigan 48824

**Keywords:** *Fragaria*, Fusarium wilt, strawberry, innate immunity, polyploid

## Abstract

*Fusarium* wilt, a soil-borne disease caused by the fungal pathogen *Fusarium oxysporum* f. sp. *fragariae*, threatens strawberry (*Fragaria* × *ananassa*) production worldwide. The spread of the pathogen, coupled with disruptive changes in soil fumigation practices, have greatly increased disease pressure and the importance of developing resistant cultivars. While resistant and susceptible cultivars have been reported, a limited number of germplasm accessions have been analyzed, and contradictory conclusions have been reached in earlier studies to elucidate the underlying genetic basis of resistance. Here, we report the discovery of *Fw1*, a dominant gene conferring resistance to *Fusarium* wilt in strawberry. The *Fw1* locus was uncovered in a genome-wide association study of 565 historically and commercially important strawberry accessions genotyped with 14,408 SNP markers. Fourteen SNPs in linkage disequilibrium with *Fw1* physically mapped to a 2.3 Mb segment on chromosome 2 in a diploid *F. vesca* reference genome. *Fw1* and 11 tightly linked GWAS-significant SNPs mapped to linkage group 2C in octoploid segregating populations. The most significant SNP explained 85% of the phenotypic variability and predicted resistance in 97% of the accessions tested—broad-sense heritability was 0.96. Several disease resistance and defense-related gene homologs, including a small cluster of genes encoding nucleotide-binding leucine-rich-repeat proteins, were identified in the 0.7 Mb genomic segment predicted to harbor *Fw1*. DNA variants and candidate genes identified in the present study should facilitate the development of high-throughput genotyping assays for accurately predicting *Fusarium* wilt phenotypes and applying marker-assisted selection.

Cultivated strawberry (*Fragaria* × *ananassa* Duchesne ex Rozier) plant health and yield are adversely impacted by several soil-borne diseases (Maas 1998). One of the greatest threats to strawberry production worldwide has been *Fusarium* wilt, a soil-borne disease caused by the fungal pathogen *Fusarium oxysporum* f. sp. *fragariae* (Winks and Williams 1965; Okamoto *et al.* 1970; Mena *et al.* 1975; Castro-Franco and Davalos-Gonzalez 1990; Huang *et al.* 2005, Abdet-Sattar *et al.* 2008, Arroyo *et al.* 2009; Koike *et al.* 2009; Gordon 2017; Henry *et al.* 2017). Historically, strawberry fruit and nursery stock growers have relied on powerful soil fumigants to suppress *F. oxysporum* f. sp. *fragariae* and other soil-borne pathogens, allowing for monocultures or very tight crop rotation cycles (Goodhue *et al.* 2005; Lloyd and Gordon 2016; Tourte *et al.* 2016). Because fruit and nursery production are typically concentrated in unique coastal and high-elevation environments in California, the availability of land for crop rotations is limited, often necessitating continuous cropping (Guthman 2016).

Until 2005, the most widely used soil fumigant in strawberry production was methyl bromide (MeBr), an ozone-layer depleting chemical compound banned by a global treaty enacted to protect Earth’s atmosphere (Montzka *et al.* 2003; Velders *et al.* 2007). MeBr was commonly applied in combination with the fumigant chloropicrin. This combination was highly effective and provided growers with predictable control of fungi and weeds (Lloyd and Gordon 2016). The efficacy of fumigation with MeBr and chloropicrin, coupled with sophisticated production practices, and the introduction of increasingly higher yielding cultivars, doubled per-acre yields and increased production 10-fold in the US from 1970 to 2016 (United States Department of Agriculture (USDA) 2017a,b). The progressive phaseout of MeBr, pursuant to the Montreal Protocol (Montzka *et al.* 2003; Velders *et al.* 2007), concluded in 2016, the last year exemptions granted for strawberry growers (Downing 2016). The fumigant mixtures available to growers have failed to effectively suppress populations of soil-borne pathogens compared to previous MeBr mixtures. Thus far, chemical and non-chemical alternatives as effective as MeBr + chloropicrin fumigation have not emerged (Downing 2016; Goodhue *et al.* 2016; Guthman 2016; Guthman and Brown 2016a,b).

*Fusarium* wilt was first discovered in strawberry in California in 2006 (Koike *et al.* 2009), in fields where fully effective fumigation practices had not been used for several years in succession (Gordon *et al.* 2016). The pathogen has since become more widespread and currently appears to be found throughout the state (Henry *et al.* 2017). Consequently, *Fusarium* wilt has become an increasingly serious threat to strawberry production in California, as in many other parts of the world (Paynter *et al.* 2014; Koike and Gordon 2015; Paynter *et al.* 2016; Gordon *et al.* 2016; Henry *et al.* 2017). The development and deployment of resistant cultivars might be the only strategy that can provide reliable and adequate control of *Fusarium* wilt in strawberry.

The identification of genes conferring resistance to *Fusarium* wilt and the development of *Fusarium* wilt resistant cultivars has a long history in tomato (*Solanum lycopersicum* L.) and other economically important plants (Michielse and Rep 2009; Takken and Rep 2010). Several novel resistance (*R*) genes have been discovered, cloned, functionally characterized, and deployed in tomato (Ori *et al.* 1997; Houterman *et al.* 2008; Takken and Rep 2010; Andolfo *et al.* 2014; Gonzalez-Cendales *et al.* 2016; Catanzariti *et al.* 2017). The most extensively studied resistance (*R*) genes belong to super-families with highly conserved nucleotide binding (NB) and C-terminal leucine-rich repeat (LRR) domains (Martin *et al.* 2003; Wulff *et al.* 2011; Dangl *et al.* 2013). These have supplied several loci and alleles for developing tomato cultivars resistant to *F. oxysporum* f. sp. *lycopersici* (Andolfo *et al.* 2014; Gonzalez-Cendales *et al.* 2016; Catanzariti *et al.* 2017). While the *R*-genes described in tomato have been fairly durable, some have been overcome by the emergence of virulent races of the pathogen, the outcome of an ongoing evolutionary arms race (Anderson *et al.* 2010), which has necessitated the identification and deployment of novel *R*-genes (Houterman *et al.* 2009; Gonzalez-Cendales *et al.* 2016; Catanzariti *et al.* 2015, 2017).

Thus far, insights into the genetics of resistance to *Fusarium* wilt in strawberry have been limited. Several strawberry cultivars have been reported to be either highly susceptible or moderately to highly resistant to *F. oxysporum* f. sp. *fragariae* (Takahashi *et al.* 2003; Dávalos-González *et al.* 2004; Hutton and Gomez 2006; Takahashi *et al.* 2006; Fang *et al.* 2012a,b, 2013; Paynter *et al.* 2014; Koike and Gordon 2015; Husaini and Neri 2016; Borrero *et al.* 2017). While a full range of disease symptoms have been reported, including apparent immunity, genetic factors underlying resistance to *Fusarium* wilt have not been identified, and contradictory conclusions have been reached in previous studies (Mori *et al.* 2005; Paynter *et al.* 2014). Mori *et al.* (2005) observed the segregation of a dominant gene in a population developed from a cross between resistant (Asuka Wave) and susceptible (Sanchiigo) cultivars but observed significant phenotypic variability within resistant and susceptible classes and concluded that resistance was caused by qualitative and quantitative genetic factors. Paynter *et al.* (2014), in a study of segregating populations developed from crosses between resistant and susceptible cultivars, concluded that resistance to *Fusarium* wilt was quantitative, and reported an intermediate heritability (0.49). To explore these questions and develop insights into the genetics of resistance to *Fusarium* wilt in strawberry, we analyzed 565 germplasm accessions in the University of California, Davis (UCD) germplasm collection. Using genome-wide association and genetic mapping approaches, we identified and genetically and physically mapped a dominant gene (*Fw1*) that confers resistance to *Fusarium* wilt in strawberry. Our study was enabled by the availability of a high-density single-nucleotide polymorphism (SNP) genotyping array (Bassil *et al.* 2015; Verma *et al.* 2017) and a high-quality reference genome for woodland strawberry (*F. vesca* (A. Heller) Staudt), a diploid (2n = 2x = 14) ancestor of *F*. × *ananassa* (Edger *et al.* 2018).

## Materials and Methods

### Plant Material

The UC-Davis Strawberry Germplasm Collection was the source of the plant material investigated in our studies. We selected 565 *F*. × *ananassa* germplasm accessions for phenotypic screening and genome-wide association studies (GWAS). These included 50 UCD cultivars released since the inception of the breeding program in 1927, and numerous previously undocumented and uncharacterized germplasm accessions representing genetic diversity in the collection (File S2). To develop populations for quantitative trait locus (QTL) mapping, S_1_ progeny were developed by self-pollinating greenhouse-grown plants of the cultivars Fronteras and Portola. These cultivars were identified as highly resistant in the GWAS. Both were self-pollinated prior to knowing if they were heterozygous or homozygous for the *Fusarium* wilt resistance gene described herein. Genotypes of the parents and grandparents (Fronteras = 04C018P004/05C165P001 and Portola = 97C093P007/97C209P001) were subsequently inferred from the haplotypes of SNPs in linkage disequilibrium with the *Fusarium* wilt resistance gene. Fruit originating from self-pollination were harvested and macerated in a pectinase solution (0.6 g/L) to separate achenes (seeds) from receptacles. S_1_ seeds were scarified with a concentrated sulfuric acid solution (36 Normal) for 14 min, rinsed in water, dried on blotter paper, and germinated at room temperature (approximately 22-24°) in June 2016. S_1_ seedlings were grown in kiln-dried artificial media (2-parts vermiculite, 1-part sand) in a shade house in Winters, CA from June to October 2016.

### Field Experiments

Germplasm accessions were phenotyped in experiments planted in the spring of 2016 and fall of 2016 in separate fields at the UC-Davis Plant Pathology Farm, Davis, CA. S_1_ populations were phenotyped in the fall-planted experiment. Neither field had ever been planted with strawberries. The soil type was a Yolo loam based on information provided by the Web Soil Survey (WSS), USDA Natural Resources Conservation Service (https://websoilsurvey.sc.egov.usda.gov/). The spring-planted field was not fumigated, whereas the fall-planted field was flat-fumigated in July 2016 with a chloropicrin-based fumigant (Pic-Clor 60, Cardinal Professional Products, Woodland, CA) at 500 lbs/acre. The fumigated field was sealed with a totally impermeable film (TIF) tarp for one week.

Entries in both experiments were arranged in balanced square lattice experiment designs with four single-plant replications per entry. The configuration was 24 × 24 (= 576 entries) in the spring-planted experiment and 31 × 31 (= 960 entries) in the fall-planted experiment. The number of entries and lattice configuration were greater in the fall-planted experiment because additional germplasm accessions were tested for a study to be reported elsewhere. The incomplete block assignments and randomizations were generated in the R-package ‘agricolae’ (De Mendiburu 2015). Bare-root plants were planted in 15.25 cm high raised beds in a single row with 30.5 cm spacing between plants and 76.2 cm spacing between beds center-to-center. We installed drip irrigation and covered the beds with black plastic mulch prior to planting. These experiments were sub-surface drip-irrigated as needed to maintain adequate soil moisture. Fertilization was done via injection through the drip system with approximately 198 kg/ha of nitrogen applied over the growing season.

The spring-planted GWAS experiment was phenotyped in the spring and summer of 2016, whereas the fall-planted GWAS experiment was phenotyped in the spring and summer of 2017. For the spring-planted 2016 field experiment, clonal propagules were produced from stolons of mother plants grown in a low-elevation (41m) nursery in Winters, CA, harvested in January 2016, and stored at -3°. The plants were removed from the freezer and stored at 3.5° for one week prior to planting in the field in April 2016. For the fall-planted 2016-2017 field experiment, clonal propagules of germplasm accessions were produced from stolons of mother plants grown in a high-elevation (1,294 m) nursery in Dorris, CA. The mother plants were harvested from a low-elevation field nursery in January 2016, stored at -3°, and planted in April 2016 at the high-elevation nursery. Clonal propagules were harvested in October 2016 and stored at 3.5° for two weeks before pathogen-inoculation and planting. For S_1_ populations phenotyped in the fall-planted 2016-2017 experiment, seeds were germinated in June 2016, transplanted to peat pellets, and grown in a shade house in Winters, CA before being pathogen-inoculated and transplanted to the field in October 2016.

### Disease Resistance Phenotyping

Seventeen-week-old S_1_ seedlings and clonal propagules of the germplasm accessions were inoculated with a virulent isolate of *F. oxysporum* f. sp. *fragariae* (AMP132) immediately before planting. To produce spores (inoculum), the pathogen was grown on potato dextrose agar (PDA) under continuous fluorescent lighting at room temperature for 30 days, as described by Gordon *et al.* (2016). Spores were freed from the surface by adding sterile deionized (DI) water with 0.5% potassium chloride (KCl) to the growth plates and scraping the edge of the media with a sterile glass slide. The resulting suspension was filtered through two layers of sterilized cheesecloth. Spore densities were estimated using a hemocytometer. Spore suspensions were diluted with 0.1% water agar to a final density of 5 × 10^6^ spores/ml. Spore inoculum was prepared one day prior to planting and stored at 4° overnight. The suspension was shaken to re-suspend spores before inoculation. Seedlings and bare-root plants were dipped in the spore suspension and immediately planted.

For each study, phenotyping was initiated as soon as symptoms appeared and continued on a weekly basis in the spring of 2016 (4-9 weeks post-inoculation) and bi-weekly basis in the spring of 2017 (26-36 weeks post-inoculation). We utilized the progression of disease symptoms as a guide for both initiating and terminating phentoyping. Symptoms had fully progressed and were most extreme among resistant and susceptible checks in the final time point in each year (9-weeks post-inoculation in 2016 and 36-weeks post-inoculation in 2017). We used a previously described 1-5 rating scale to phenotype disease symptoms, where symptomless plants were scored as 1, stunted plants were scored as 2, chlorotic plants were scored as 3, wilting plants were scored as 4, and plants killed by the pathogen were scored as 5 (Gordon *et al.* 2016; Henry *et al.* 2017).

### DNA Marker Genotyping

For DNA isolation, newly emerging leaves were harvested from field grown plants of the germplasm accessions and shade house-grown seedlings of S_1_ populations. Leaf tissue was placed into 1.1 ml tubes, freeze-dried in a Benchtop Pro (VirTis SP Scientific, Stone Bridge, NY), and ground using stainless steel beads in a Mini 1600 (SPEX Sample Prep, Metuchen, NJ). Genomic DNA (gDNA) was extracted from powdered leaf samples using the E-Z 96 Plant DNA Kit (Omega Bio-Tek, Norcross, GA, USA) according to the manufacturer’s instructions. To enhance the quality of the DNA and reduce polysaccharide carry-through, the protocol was modified with a Proteinase K treatment, a separate RNase treatment, an additional spin, and heated incubation steps during elution. DNA quantification was performed using Quantiflor dye (Promega, Madison, WI) on a Synergy HTX (Biotek, Winooski, VT).

SNP genotyping with the Affymetrix IStraw35 Axiom Array (Bassil *et al.* 2015; Verma *et al.* 2017) was performed by Affymetrix (Santa Clara, CA) on a GeneTitan HT Microarray System using gDNA samples that passed quality and quantity control standards. SNP genotypes were automatically called with the Affymetrix Axiom Analysis Suite software (v1.1.1.66, Affymetrix, Santa Clara, CA). Samples with a call-rate greater than 94% were retained. The quality metrics output by the Affymetrix Axiom Analysis Suite, custom R scripts, and the R-package ‘SNPRelate’ (Zheng *et al.* 2012) were utilized to filter SNPs; 14,408 SNPs with high-quality bi-allelic clusters and < 5% missing data were selected for subsequent analyses. The R-packages ‘SNPRelate’ (Zheng *et al.* 2012) and ‘GWASTools’ (Gogarten *et al.* 2012) were used to generate genotypic input files for GWAS from raw genotyping reads.

### Genome-Wide Association Study

Type III analysis of variance (ANOVA) was performed using a mixed linear model for the lattice experiment design with incomplete and complete blocks as random effects and entries as fixed effects. Statistical analyses were performed using the R-packages ‘lme4’ and ‘car’ (Fox and Weisberg 2011; Bates *et al.* 2015). The recovery of intra-block error information from the lattice experiment designs was negligible and failed to increase efficiency relative to randomized complete block (RCB) experiment designs; hence, we utilized linear models for RCB experiment designs for subsequent analyses. Least square means for entries were estimated using the R-package ‘lsmeans’ with complete blocks as a random effect and entries as a fixed effect (Lenth 2016) and were subsequently used as phenotypic input for GWAS. REML variance component and broad-sense heritabilities (Lynch and Walsh 1998) were estimated using the R-package ‘lme4’ (Bates *et al.* 2015), with entries, complete blocks, and years as random effects.

Because the germplasm collection we studied included numerous closely related individuals, we investigated and accounted for population structure using principal components analysis in related samples (PCAiR) with ‘GENESIS’ (http://bioconductor.org/packages/release/bioc/vignettes/GENESIS/inst/doc/pcair.html; Conomos *et al.* 2015, 2016; Conomos and Thornton 2016). The *p*-value inflation factors (λ), ignoring population structure, were 3.09 for the 2016 and 3.69 for the 2017 GWAS experiments (Clayton 2015). We subsequently used the first three principal components (PCs) from PC-AiR as input for calculating the kinship matrix, which was done using PC-relate in ‘GENESIS’ (Conomos and Thornton 2016). The resultant kinship matrix was used in a mixed linear model GWAS analysis, assuming a Gaussian distribution of the dependent variable. Wald tests were performed as implemented in ‘GENESIS’ using default parameters (Conomos and Thornton 2016). Because an octoploid reference genome was unavailable, we utilized a diploid reference genome for *F. vesca* (Edger *et al.* 2018) for GWAS, plotting *p*-values against physical positions (Mb). SNP probe sequences from the Affymetrix IStraw35 Axiom Array (Bassil *et al.* 2015; Verma *et al.* 2017) were physically mapped to the diploid reference genome using the Burrows-Wheeler Aligner (BWA v.0.7.15; Li and Durbin 2009). The ancestry-adjusted *p*-value inflation factors were 0.75 for the 2016 and 0.85 for the 2017 GWAS experiments, which suggested that the population structure corrections in the mixed linear model GWAS were effective (Hinrichs *et al.* 2009).

### Genetic and Quantitative Trait Locus Mapping

Because the parents and grandparents of the S_1_ populations were outbred, genetic mapping was performed using the full-sib mapping algorithm of JoinMap 4.1 (van Ooijen 2006), which utilizes the general maximum-likelihood (ML) algorithm of Wu *et al.* (2002) for simultaneously estimating linkage and linkage phases in full-sib families. Because we selfed a single individual descended from two outbred parents (grandparents of the S_1_ offspring), heterozygous loci were expected to segregate 1 AA: 2 AB: 1 BB in the Fronteras and Portola S_1_ populations, where A is the allele inherited from one grandparent and B is the allele inherited from the other grandparent. S_1_ individuals were genotyped with the Affymetrix IStraw35 Axiom Array (Bassil *et al.* 2015; Verma *et al.* 2017). SNPs that produced co-dominant (bi-allelic) segregation patterns identified using the Affymetrix Axiom Analysis Suite were selected for subsequent analyses. For genetic mapping, we identified and selected 5,673 SNPs in the Fronteras S_1_ population and 7,345 SNPs in the Portola S_1_ population. Numerous SNPs were in complete LD across the genome. To reduce the dimensions of the data and more robustly order loci, co-segregating SNPs were assigned to bins and one SNP from each bin was selected for inclusion in the analysis. Once linkage phases were estimated, SNPs were recoded according to the inferred linkage phase, analogous to an F_2_ population developed from a cross between inbred parents. Loci were grouped using a minimum likelihood odds (LOD) threshold of 8.0 and ordered using the multi-point ML algorithm in JoinMap 4.1 with default parameters and three rounds of locus ordering (van Ooijen 2006). Genetic distances were calculated using the Kosambi mapping function. By cross-referencing previously mapped iStraw35 and iStraw 90 SNPs (Verma *et al.* 2017), linkage groups identified in the present study were aligned with 28 linkage groups previously described by Bassil *et al.* (2015) and Mangandi *et al.* (2017). The linkage group numbers and orientations in Bassil *et al.* (2015) and Mangandi *et al.* (2017) trace their origin to van Dijk *et al.* (2014).

We assigned S_1_ offspring to resistant and susceptible phenotypic classes and tested the hypothesis of the segregation of a single gene using standard goodness-of-fit statistics. Offspring with *Fusarium* wilt scores < 2.5 were classified as resistant, whereas offspring with *Fusarium* wilt score ≥ 2.5 were classified as susceptible. The observed segregation ratios were tested for goodness-of-fit to the expected segregation ratio of three resistant to one susceptible using Chi-square statistics with the R-function ‘chisq.test’.

Linkage groups were scanned for quantitative trait loci (QTL) using the interval mapping function in MapQTL 6 (van Ooijen 2009). Several tightly linked SNPs on linkage group 2C, previously identified by GWAS, co-segregated with a QTL that was targeted in subsequent analyses. Significant SNP loci in the haploblock were individually used as fixed effects (independent variables) in linear model analyses to estimate additive (*a*) and dominance (*d*) effects, degree of dominance (*d*/*a*), and the proportion of the phenotypic variance associated with the additive and dominance effects of the SNP locus (Falconer and Mackay 1996; Lynch and Walsh 1998). SNPs were physically mapped in the diploid reference genome (Edger *et al.* 2018). We used linkage phases of SNP markers to infer the haplotypes of the parents (Fronteras and Portola). The inferred haplotypes were supported by the three-generation pedigree of Fronteras. The 05C165P001 parent of Fronteras was susceptible to *Fusarium* wilt and homozygous for the eight most significant SNPs, whereas the 04C018P004 was resistant to *Fusarium* wilt and heterozygous for the eight most significant SNPs (File S2).

### Data Availability

All data required to replicate the analyses are available as supplements cited in-text or in Supplemental Data Files 1-4. Supplemental Data Files 1 and 2 contain the raw genotypic data for the germplasm accessions and mapping populations, respectively. Supplemental Data File 3 provides additional information regarding SNP nomenclature, alleles, and genomic locations. Supplemental Data File 4 provides the raw phenotypic (disease symptom) scores for every time point in 2016 and 2017 studies. Supplemental material available at Figshare: https://doi.org/10.25387/g3.6007715.

## Results

**Figure 1:**
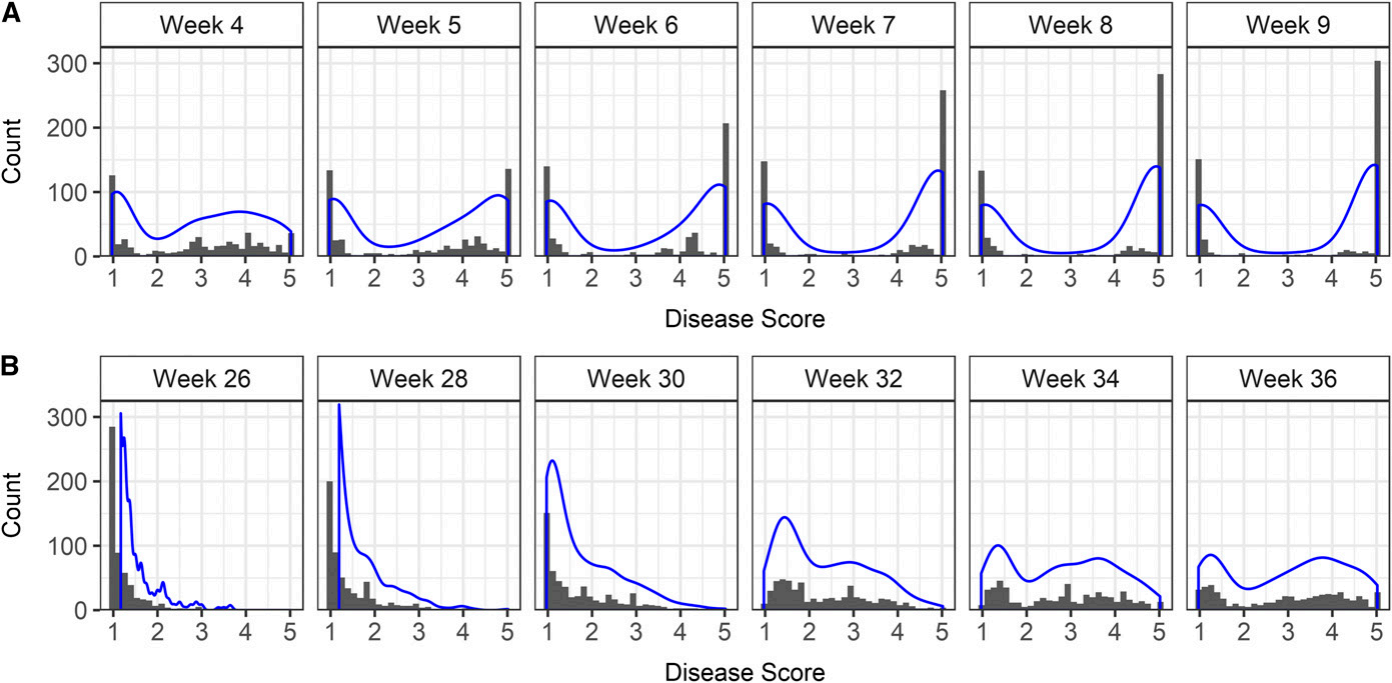
Phenotypic distributions for resistance to *Fusarium* wilt in a genome-wide association study (GWAS) in strawberry. Histograms are shown for phenotypes observed in (A) 2016 and (B) 2017 field experiments in Davis, California among 565 strawberry germplasm accessions artificially inoculated with isolate AMP132 of *Fusarium oxysporum* f. sp. *fragariae*. Phenotypes were observed four to nine weeks post-inoculation in 2016 and 26 to 36 weeks post-inoculation in 2017. Least square means were estimated from four clonal replicates per entry with entries arranged in a square lattice experiment design. Disease scores ranged from 1 to 5, where 1 = healthy and 5 = dead.

**Figure 2:**
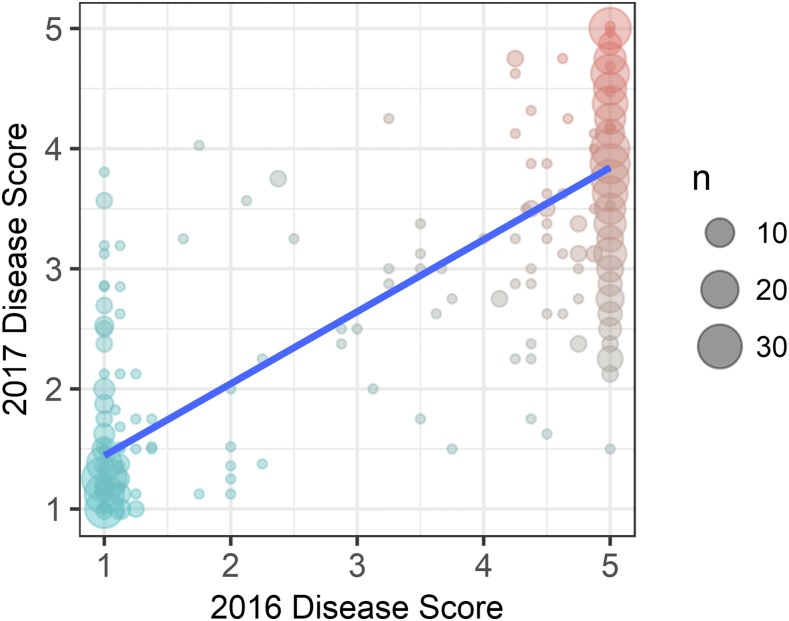
Phenotypic correlation (Pearson’s r = 0.84, *P* < 0.001) between years for *Fusarium* wilt resistance phenotypes in strawberry. A fitted linear regression is shown in blue. *Fusarium* wilt resistance was phenotyped in 2016 and 2017 field experiments in Davis, California among 565 strawberry germplasm accessions artificially inoculated with isolate AMP132 of *Fusarium oxysporum* f. sp. *fragariae*. The phenotypes shown were observed nine weeks post-inoculation in 2016 (x-axis) and 36 weeks post-inoculation in 2017 (y-axis).

One-third of the germplasm accessions screened for resistance to the AMP132 isolate of *F. oxysporum* f. sp. *fragariae* were symptomless and classified as resistant (Figure 1). Apart from a small number of germplasm accessions with intermediate symptoms (<2% of those studied), nearly two-thirds developed severe vascular wilt symptoms, including stunting, chlorosis, wilting, browning, and dieback (File S2). To study the progression of disease symptoms and quantify changes in phenotypic distributions over time, plants were phenotyped over six weeks in 2016 and 10 weeks in 2017 (27,168 phenotypic observations were collected over the course of the study). As predicted *a priori*, the shapes of the phenotypic distributions changed as symptoms progressed and disease severity increased (Figure 1). Symptoms developed more rapidly and phenotypic differences were more extreme in 2016 than 2017. The final 2016 phenotypic distribution (9 weeks post-inoculation) was bi-modal with strong separation between resistant and susceptible accessions, whereas the final 2017 phenotypic distribution (36 weeks post-inoculation) was flatter with weaker separation between resistant and susceptible accessions (Figure 1; File S2). Symptom development was less severe for several susceptible accessions in 2017 than 2016. While the accession by year interaction was highly significant as a result (*P* < 0.001; File S1), the phenotypic correlation between years was positive and highly significant (*r* = 0.84; *P* < 0.001; Figure 2), broad-sense heritabilities exceeded 90% (*H*^2^ = 0.98 in 2016, 0.90 in 2017, and 0.96 across years), and the classification of accessions as resistant or susceptible was highly consistent over years (Figure 2; File S2).

**Figure 3:**
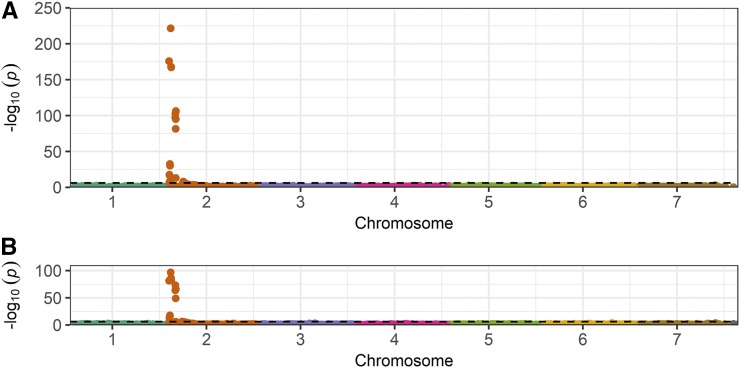
Genome-wide association study for resistance to *Fusarium* wilt in octoploid strawberry using chromosome positions from the diploid (x = 7) *F. vesca* reference genome (Edger *et al.* 2018). Manhattan plots are for phenotypes observed in 2016 (A) and 2017 (B) experiments. The horizontal dashed line identifies a 0.01 Bonferroni-corrected significance threshold.

**Figure 4:**
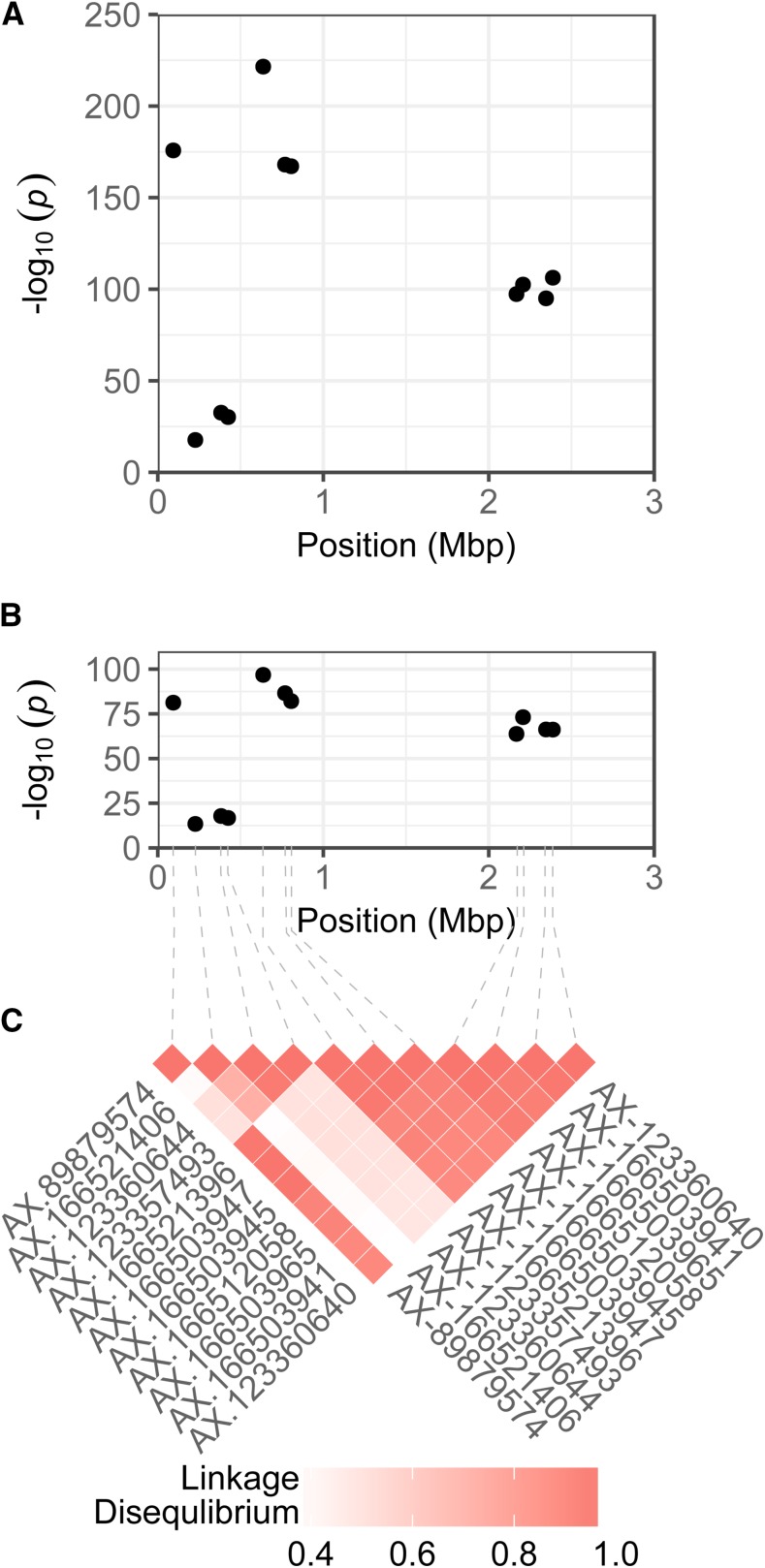
SNPs in linkage disequilibrium with a *Fusarium* wilt resistance gene (*Fw1*) that were genetically mapped to chromosome 2C in octoploid strawberry. Manhattan plots are shown for phenotypes observed in 2016 (A) and 2017 (B) GWAS experiments with -log_10_ *p*-values for nine SNPs plotted against chromosome positions in a diploid (x = 7) *F. vesca* reference genome (Edger *et al.* 2018). Pairwise marker linkage disequilibrium statistics are shown for the GWAS panel (C).

Genome-wide association studies were conducted using 14,408 SNPs mapped against chromosome positions in a diploid (2n = 2x = 14) *F. vesca* reference genome (Edger *et al.* 2018). GWAS identified 14 SNPs in a 2.3 Mb genomic segment on the upper arm of chromosome 2 that were in linkage disequilibrium with *Fusarium* wilt resistance phentoypes (Figure 3; File S2). The -log_10_ *p*-values for significant SNPs greatly exceeded conservative genome-wide statistical significance thresholds, ranging from 9.18 × 10^−9^ for AX-123360644 in the 2017 experiment to 2.95 × 10^−222^ for AX-166521396 in the 2016 experiment (Figure 3; File S2). Significant SNPs were not identified elsewhere in the genome. When genomic locations were examined in greater depth, the 14 SNPs were discovered to have mapped to 0.71 Mb and 0.22 Mb genomic segments separated by a 1.36 Mb segment that was devoid of significant SNPs (Figure 4; File S2). The most significant SNP in the study (AX-166521396) was located in the upper 0.7 Mb genomic segment (Figure 4; File S2).

**Figure 5:**
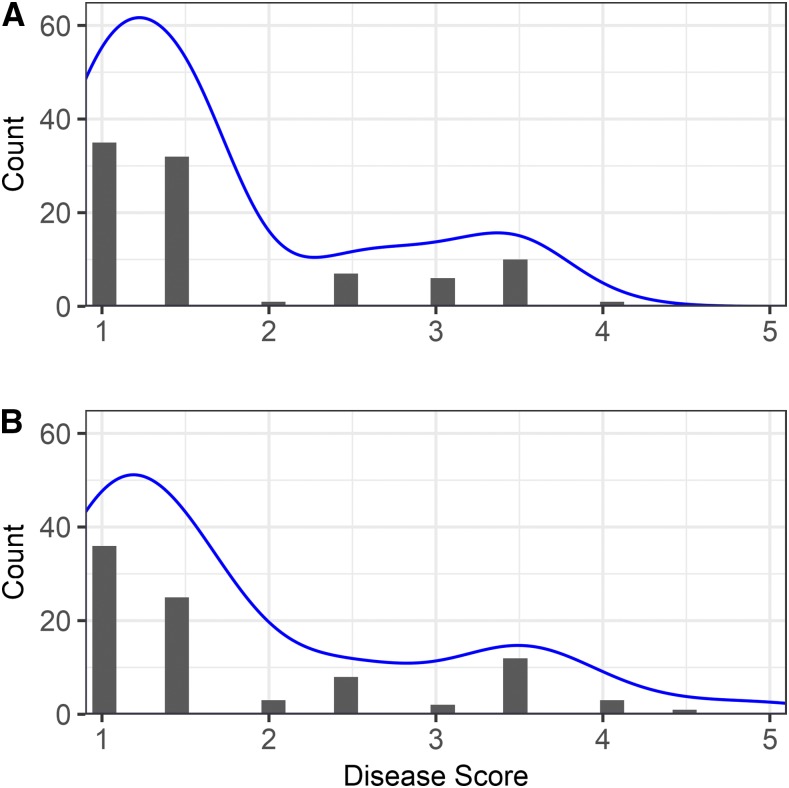
Distributions for *Fusarium* wilt resistance phenotypes observed in octoploid segregating populations. Histograms are shown for *Fusarium* wilt resistance phenotypes in segregating populations developed by self-pollinating the *F*. × *ananassa* cultivars (A) Fronteras and (B) Portola. Parents (Fronteras and Portola) and grandparents (04C018P004/05C165P001 and 97C093P007/97C209P001) of the S_1_ populations and 93 S_1_ individuals from each population were artificially inoculated with *Fusarium oxysporum* f. sp. *fragariae* isolate AMP132. Phenotypes were observed 36 weeks post-inoculation in a 2017 field experiment in Davis, California.

From the strength of the GWAS signal and short physical distance spanned by significant SNPs on chromosome 2 in the diploid reference genome (Figures 3-4), we hypothesized that the SNP haploblock was in linkage disequilibrium with a gene conferring resistance to *Fusarium* wilt on a chromosome 2 homeolog in the octoploid genome. To investigate this, genetic and QTL mapping studies were conducted in S_1_ populations developed by self-pollinating cultivars (Fronteras and Portola) inferred to be heterozygous for the hypothesized resistance gene (File S2). The phenotypic distributions for both S_1_ populations were bi-modal with fairly distinct separation between resistance and susceptible classes (Figure 5). For hypothesis testing, progeny with phenotypic scores < 2.5 were classified as homozygous or heterozygous resistant (*R*_), whereas progeny with phenotypic scores ≥ 2.5 were classified as homozygous susceptible (*rr*). Using these classifications, the observed phenotypic ratios were not significantly different from 3 *R*_: 1 *rr*, the phenotypic ratio expected for the segregation of a dominant gene. We observed 68 *R*_: 24 *rr* among Fronteras S_1_ progeny (χ^2^ = 0.06; *P* = 0.81) and 64 *R*_: 28 *rr* among Portola S_1_ progeny (χ^2^ = 1.45; *P* = 0.23). Shifting the threshold upward to 3 or downward to 2 did not change the statistical inference.

**Figure 6:**
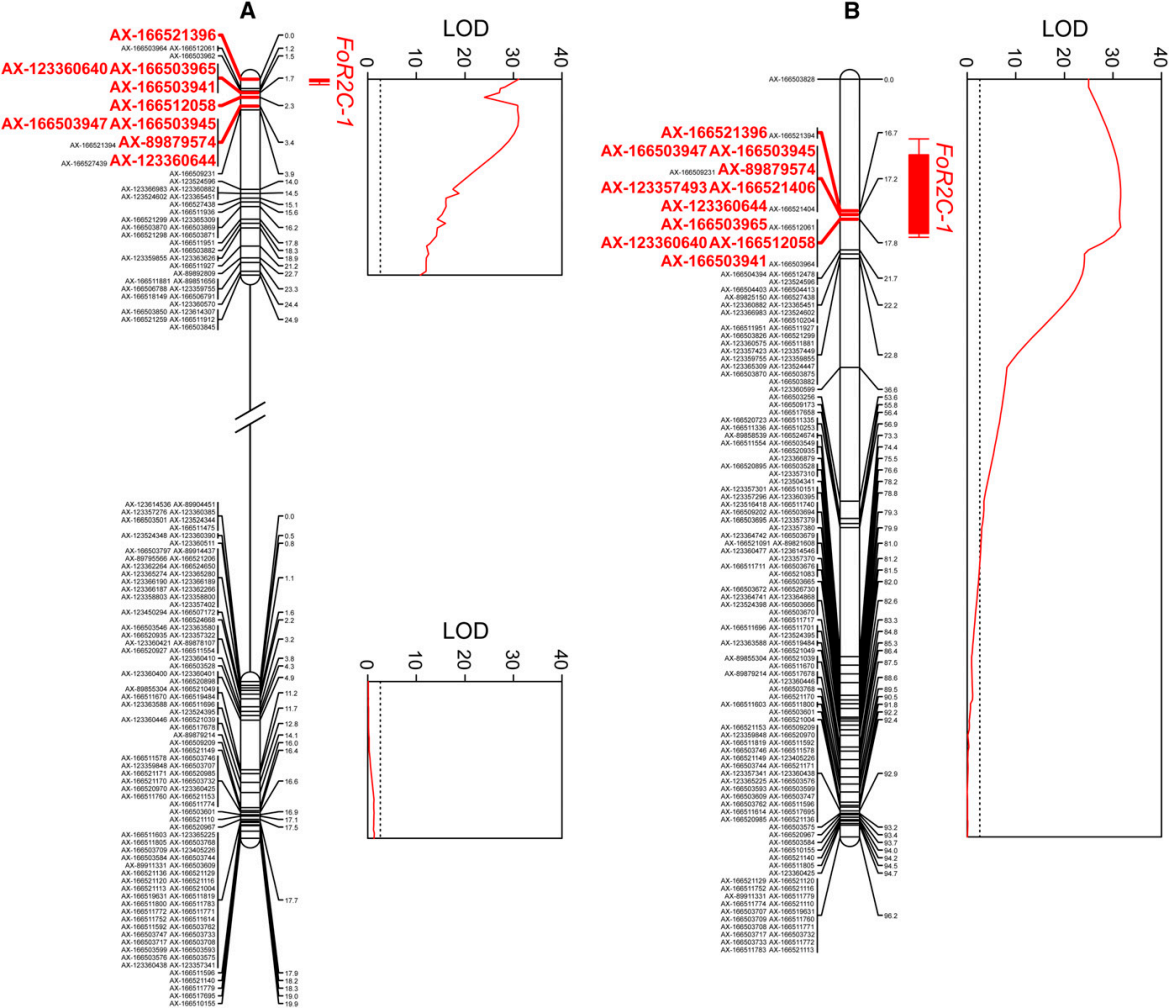
Genetic mapping of a *Fusarium* wilt resistance gene (*Fw1*) in octoploid segregating populations. Likelihood-odds (LOD) statistics and linkage group positions (cM) are shown for a quantitative trait locus (QTL) for *Fusarium* wilt resistance identified by interval mapping in (A) Portola and (B) Fronteras S_1_ mapping populations. The parents (Fronteras and Portola) and grandparents (04C018P004/05C165P001 and 97C093P007/97C209P001) of the S_1_ populations and 93 S_1_ individuals from each population were genotyped with the iStraw35 SNP array and artificially inoculated with isolate AMP132 of *Fusarium oxysporum* f. sp. *fragariae* at planting. Phenotypes were observed 36 weeks post-inoculation in a 2017 field experiment in Davis, California. The *Fw1* QTL mapped to identical locations on the upper arm of chromosome 2C in both populations. One- and two-LOD confidence intervals are shown. Highlighted SNPs (bold red) were significant in genome-wide association studies.

Using a high-density SNP array (Bassil *et al.* 2015; Verma *et al.* 2017), 5,673 co-dominant SNPs were genotyped and mapped in the Fronteras S_1_ population, whereas 7,345 co-dominant SNPs were genotyped and mapped in the Portola S_1_ population (Supplemental Files 3-4). Because SNP marker densities were low in several genomic regions, the number of linkage groups (40 in the Fronteras S_1_ and 50 in the Portola S_1_ population) exceeded the haploid chromosome number (28). Nevertheless, by cross-referencing SNPs previously mapped by Mangandi *et al.* (2017), sub-linkage groups were aligned and oriented with 28 previously identified *F*. × *ananassa* linkage groups numbered using the nomenclature of van Dijk *et al.* (2014). Using interval mapping, a single large-effect QTL was identified on linkage group 2C in both S_1_ populations (Figure 6). Eleven of the 14 GWAS-identified SNPs segregated, mapped to a short interval on the upper arm of linkage group 2C, and co-segregated with the QTL (Figure 6; Supplemental Files 3-4). Two of the 11 SNPs (AX-166521406 and AX-123357493) only segregated in the Fronteras S_1_ population. Additive and dominance effects for individual SNPs were highly significant (*P* < 0.001) and nearly identical across the linkage group 2C haploblock within each population (File S3). The additive and dominance effects for the AX-166521396 SNP locus were -0.85 and -0.86 in the Fronteras S_1_ population and -1.12 and -0.94 in the Portola S_1_ population (File S3). The AX-166521396 SNP explained 85% of the phenotypic variation for resistance to *Fusarium* wilt in both S_1_ populations (Figure 6; File S3). Significant QTL were not identified elsewhere in the genome (File S4). The QTL was completely dominant in one population and nearly completely dominant in the other—the degree of dominance (|d/a|) for the AX-166521396 SNP was 1.01 in the Fronteras and 0.84 in the Portola S_1_ population (File S3). We concluded that the QTL was caused by the segregation of a dominant gene conferring resistance to *Fusarium* wilt, hereafter identified as *Fw1*. One recombinant individual was observed between the upper and lower SNP haploblocks among 186 S_1_ individuals. The recombinant individual (16S408P105) was susceptible to *Fusarium* wilt, homozygous for the susceptible haplotype in the upper haploblock, and heterozygous for the resistant haplotype in the lower haploblock; hence, *Fw1* appears to be located upstream of the lower haploblock within or near the upper haploblock harboring the AX-166521396 SNP locus (Figures 4 and 6).

**Figure 7:**
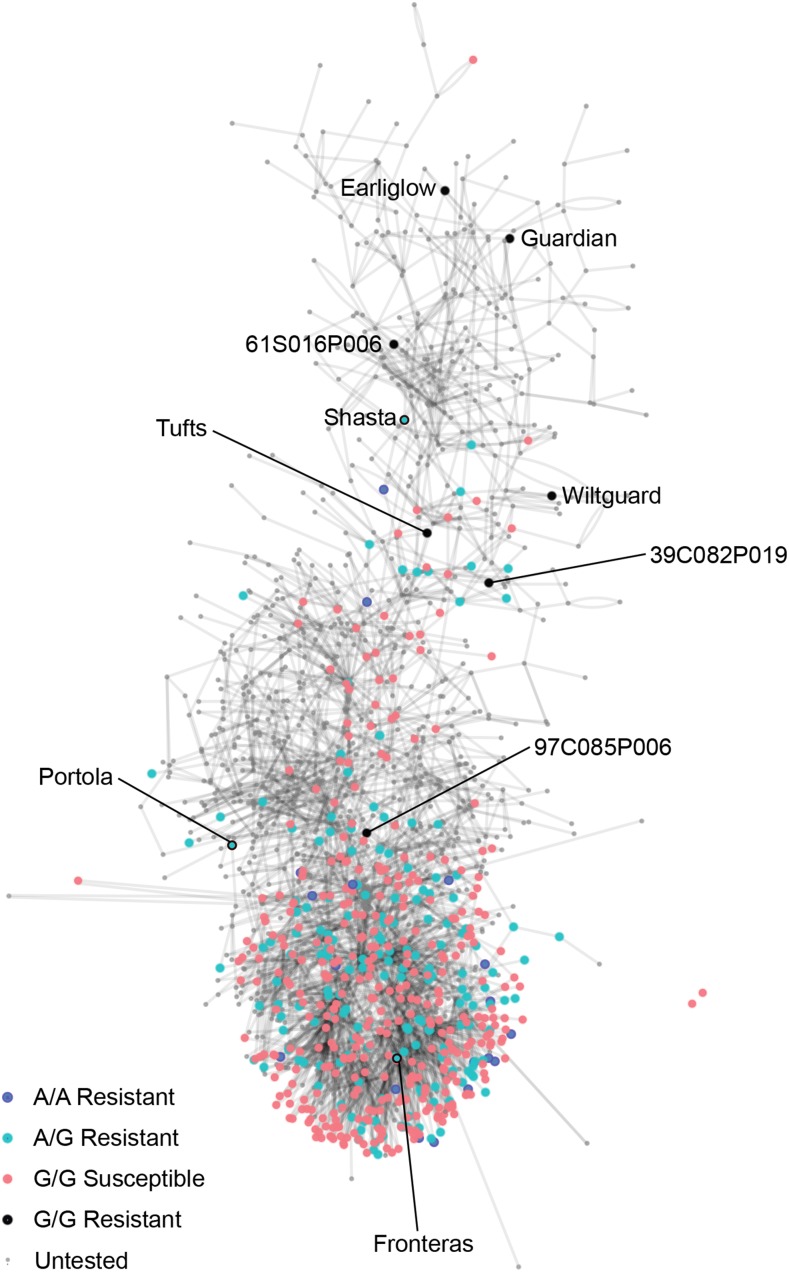
Pedigree network for 1,663 *F*. × *ananassa* germplasm accessions with birth years ranging from 1814 to 2012. The north to south orientation of the network is approximately chronological. The pedigree records are supplied in Supplemental File 5. Nodes represent germplasm accessions, whereas connecting lines represent first-degree relatives (parent-offspring). The color of the node signifies a combination of the *Fusarium* wilt resistance phenotype and the AX-166521396 SNP marker genotype for 565 germplasm accessions. The other 1,098 germplasm accessions in the pedigree network were untested (small light gray nodes). The AX-166521396 SNP marker was in linkage disequilibrium with the *Fw1* gene conferring resistance to *Fusarium* wilt. The A allele was associated with the resistant allele (*Fw1*), whereas the G allele was associated with the susceptible allele (*fw1*). AX-166521396 SNP marker genotypes predicted *Fusarium* wilt resistance phenotypes in 97% of the germplasm accessions tested: most A/A and A/G genotypes were resistant (blue and cyan filled circles, respectively), whereas most G/G genotypes were susceptible (salmon filled circles). Seven G/G genotypes were resistant and predicted to carry novel *Fusarium* wilt resistance genes (black filled circles).

Haplotypes for SNPs in linkage disequilibrium with *Fw1* were inferred from pedigree records and linkage phases in segregating populations (File S2). The haplotype associated with the resistant *Fw1* allele was observed in 161 accessions (28.4%), whereas the haplotype associated with the susceptible *Fw1* allele was observed in 368 accessions (65.0%). Within the upper haploblock, which harbors AX-166521396, the resistant haplotype was observed in 189 accessions (33.4%), whereas the susceptible haplotype was observed in 370 accessions (65.4%). To retrace the breeding history of *Fw1*, we assembled and analyzed pedigree records for *F*. × *ananassa* germplasm accessions originating between 1814 and 2014 (Figure 7; File S5). The earliest sources of the resistant *Fw1* allele among the UCD germplasm accessions tested were cultivars developed in 1935—Solana (PI 551665) and Shasta (PI 551663)—both of which were heterozygous (Figure 7). Resistant *Fw1* homozygotes were rare in the germplasm studied (<3% of the accessions). Soquel (PI 666602) was the only homozygous resistant cultivar out of 50 tested (Figure 7; File S2).

Of 11 genetically mapped SNPs in linkage disequilibrium with *Fw1* on linkage group 2C (Figure 6), eight accurately predicted phenotypes in 93.8–97.3% of the germplasm accessions tested (Figure 4; Supplemental Files 2 and 6). The A allele for the most predictive SNP (AX-166521396) was associated with the resistant *Fw1* allele, had a frequency of 0.18 in the UCD germplasm collection, and was homozygous (A/A) or heterozygous (A/G) in 190 accessions (Figure 7; File S6). The resistant *Fw1* allele appears to have survived by chance and to be randomly distributed over generations and pedigrees (Figure 7).

AX-166521396 accurately predicted *Fusarium* wilt resistance phenotypes in 97.3% of the germplasm accessions tested in the 2016 experiment: one out of 16 accessions with the A/A genotype and three out of 174 accessions with the A/G genotype were susceptible, whereas seven out of 371 accessions with the G/G genotype were resistant. The seven outliers (resistant accessions with G/G genotypes) included the cultivars Guardian (PI 551407), Wiltguard (52C016P007), Earliglow (PI 551394), and Tufts (63C120P001) and UCD germplasm accessions 39C082P019, 61S016P006, and 97C085P006 (Figure 7; File S2). Because these accessions were both highly resistant and homozygous for the susceptible *Fw1* allele (G/G), we suspect that they harbor novel *Fusarium* wilt resistance genes. These germplasm accessions, with one exception (97C085P006), originated between 1939 and 1975, upstream of apparent bottlenecks in the UCD breeding program (Figure 7).

To identify candidate genes for *Fw1*, we examined gene annotations in the 2.3 Mb segment spanned by GWAS-significant SNPs: 93,425 to 2,387,499 bp on chromosome 2 in the diploid reference genome (Edger *et al.* 2018; Supplemental Files 2 and 7). Several recurrent defense-related genes reside in the target segment (File S7), including several with conserved domains common to disease resistance genes in plants, *e.g.*, “extracellular membrane-anchored leucine-rich repeat (LRR) receptor-like proteins” and “nucleotide binding LRR proteins” (Martin *et al.* 2003; Chisholm *et al.* 2006; Wulff *et al.* 2011; Dangl *et al.* 2013). The strongest candidates for *Fw1* are Toll/interleukin-1 receptor (TIR) NB-LRR encoding genes (FvH4_2g00540, FvH4_2g00550, and FvH4_2g00570) found in a small cluster located between 577,691 and 606,648 bp on chromosome 2 in the *F. vesca* genome (Edger *et al.* 2018; File S7), immediately upstream of the most significant SNP marker (AX-166521396; 636,941 bp). The TAIR annotation for these genes returned RPP13, a TIR-NB-LRR encoding gene that confers resistance to downy mildew in Arabidopsis, a disease caused by the oomycete *Peronospora parasitica* (Bittner-Eddy *et al.* 1999, 2000; Rose *et al.* 2004; The Arabidopsis Information Resource (TAIR) 2015). The other defense-related genes in the target segment included glycosyl hydrolase (GH) and transferase genes (FvH4_2g00020, FvH4_2g00340, FvH4_2g00600, FvH4_2g02970), in addition to homologs of a vacuolar sorting protein (VPS52; FvH4_2g00140), Whirly (FvH4_2g00250), powdery mildew resistance 5 (PMR5; FvH4_2g02780), histidyl-tRNA synthetase (FvH4_2g00780), aspartyl-tRNA synthetase (FvH4_2g03040), and TARGET OF RAPAMYCIN (TOR; FvH4_2g03080). While none of these can be ruled out, *Fw1* has the hallmark of a gene encoding one of the well-known classes of *R*-genes that trigger innate immunity in plants (Martin *et al.* 2003; Chisholm *et al.* 2006; Wulff *et al.* 2011; Dangl *et al.* 2013).

## Discussion

We identified a dominant gene (*Fw1*) in octoploid strawberry that confers resistance to a virulent isolate of *F. oxysporum* f. sp. *fragariae* found in California (Gordon *et al.* 2016; Henry *et al.* 2017). Our findings were consistent with the hypothesis that gene-for-gene resistance to *Fusarium* wilt might be operating in strawberry, as previously suggested by Mori *et al.* (2005) in a study where a segregating population was screened for resistance to a Japanese isolate of *F. oxysporum* f. sp. *fragariae* (91060510). While Mori *et al.* (2005) concluded that resistance was caused by the segregation of both “qualitative and quantitative genes”, the evidence for the segregation of a dominant gene was compelling. Similar to Mori *et al.* (2005), we observed phenotypic variability among progeny classified as resistant or susceptible; however, the *Fw1* locus was sufficient to explain phenotypic variability for resistance to *Fusarium* wilt in our study (Figures 5-6; Supplemental Files 2-3). We found no evidence for the segregation of additional loci (Figure 3; File S4)—non-genetic variability was negligible and broad-sense heritabilities were in the 0.90 to 0.98 range, double the estimate reported by Paynter *et al.* (2014). Paynter *et al.* (2014) screened segregating progeny for resistance to a mixture of two virulent Australian isolates of *F. oxysporum* f. sp. *fragariae* (N13581 and N15309). The effectiveness of *Fw1* against these isolates and other isolates of the pathogen is unknown. Moreover, several factors, including the absence of characterized resistance genes, has precluded the assignment of isolates to races through the study of differential host-pathogens interactions. The present study opens the way to exploring the race structure of *F. oxysporum* f. sp. *fragariae* isolates by testing host differentials (Gordon and Martyn 1997; Takken and Rep 2010).

Several strawberry cultivars have been identified as resistant or susceptible to *Fusarium* wilt in previous studies conducted using a variety of screening protocols, pathogen isolates, and environments (Takahashi *et al.* 2003; Dávalos-González *et al.* 2004; Takahashi *et al.* 2006; Fang *et al.* 2012a,b, 2013; Paynter *et al.* 2014; Gordon *et al.* 2016; Husaini and Neri 2016; Borrero *et al.* 2017). The resistance classifications of the cultivars tested in our study were consistent with those previously reported. Of the 50 cultivars we screened, eight were previously screened for resistance to a mixture of four isolates of *F. oxysporum* f. sp. *fragariae* excluding AMP132, and yielded phenotypic classifications identical to those reported here for the AMP132 isolate (Gordon *et al.* 2016).

The potential for new races of *F. oxysporum* f. sp. *fragariae* to emerge in California and the durability of the *Fw1 R*-gene are uncertain. Genes that confer vertical resistance to pathogens are commonly defeated by the loss or mutation of effector alleles in the pathogen (Chisholm *et al.* 2006; Jones and Dangl 2006; Ronald and Beutler 2010; Takken and Rep 2010; Monaghan and Zipfel 2012), which may or may not play a role in *Fw1*-mediated resistance. As reported by Henry *et al.* (2017), a single lineage currently dominates populations of *F. oxysporum* f. sp. *fragariae* in California. Although *Fusarium oxysporum* formae speciales seem to evolve new races more slowly than many other pathogens, high concentrations of inoculum, human-aided dispersal, and selection pressure increase the probability that new races will emerge (Gordon and Martyn 1997; McDonald and Linde 2002a,b). As our study shows, most of the *Fusarium* wilt resistant cultivars developed by UCD over the last 90 years (30% of those tested) carry a single *R*-gene (*Fw1*)—the other 70% were susceptible to *Fusarium* wilt. With the emergence of the pathogen in California over the last decade (Koike *et al.* 2009; Koike and Gordon 2015), we expect the frequency of *Fusarium* wilt resistant cultivars to greatly increase in California, which, when coupled with shifting fumigation practices, could increase selection pressure on the pathogen. Tomato provides a model for predicting what might eventually transpire in strawberry (Takken and Rep 2010). Several structurally and functionally diverse *Fusarium* wilt resistance genes (*e.g.*, *I*, *I-2*, *I-3*, and *I-7*) have been described in tomato (Gonzalez-Cendales *et al.* 2016; Catanzariti *et al.* 2017). The tomato *I* gene (discovered in 1939) was less durable than the *I-2* gene (discovered in 1954); however, both were eventually defeated by the emergence of new *F. oxysporum* f. sp. *lycopersici* races (Bohn and Tucker 1939; Alexander and Tucker 1945; Takken and Rep 2010; Catanzariti *et al.* 2017). These *R*-genes operate by different mechanisms that appear to affect durability and collectively provide greater safety against pathogen evolution than the individual *R*-genes (Catanzariti *et al.* 2015, 2017). Consequently, tomato breeders have identified and pyramided multiple *Fusarium* wilt *R*-genes in the arms race with the pathogen (Takken and Rep 2010).

The development and deployment of *Fusarium* wilt resistant cultivars in strawberry will be critically important as the pathogen spreads and increases in importance in California and other parts of the world (Koike *et al.* 2009; Paynter *et al.* 2014; Gordon *et al.* 2016). The array-based SNP markers and candidate genes described here provide the foundation for developing high-throughput genotyping assays for the application of marker-assisted selection, which can accurately predict *Fusarium* wilt phenotypes and accelerate breeding efforts. Because the *Fw1 R*-allele was present in 97% of the resistant germplasm accessions tested, including several cultivars spanning the breeding history of strawberry in California (Figure 7; File S2), this allele is probably found in breeding programs around the world. Similar to tomato (Houterman *et al.* 2008; Gonzalez-Cendales *et al.* 2016; Catanzariti *et al.* 2015, 2017), our study suggests that multiple *Fusarium* wilt resistance genes exist in strawberry (Figure 7; Supplement File 4). These will undoubtedly be needed to slow the emergence of new races of the pathogen and should be identified and preemptively deployed to minimize genetic vulnerability in strawberry. Several important questions remain to be answered. The number of unique loci and alleles involved in resistance to *Fusarium* wilt is unknown, and the genes encoding *Fw1* and the other *R*-genes predicted by our study remain to be identified, cloned, and characterized. The identification and characterization of effector genes in *F. oxysporum* f. sp. *fragariae* will be critical for understanding the interaction between the strawberry and *F. oxysporum* f. sp. *fragariae* and the co-evolution of resistance and avirulence genes (Takken and Rep 2010).
